# Guided Bone Regeneration: CGF and PRF Combined With Various Types of Scaffolds—A Systematic Review

**DOI:** 10.1155/ijod/4990295

**Published:** 2024-12-05

**Authors:** Francesco Inchingolo, Angelo Michele Inchingolo, Giulia Latini, Elisabetta de Ruvo, Merigrazia Campanelli, Andrea Palermo, Massimo Del Fabbro, Marco Di Blasio, Alessio Danilo Inchingolo, Gianna Dipalma

**Affiliations:** ^1^Interdisciplinary Department of Medicine, Università degli Studi di Bari Aldo Moro, Bari 70124, Italy; ^2^College of Medicine and Dentistry, Birmingham B4 6BN, UK; ^3^Department of Biomedical Surgical and Dental Sciences, University of Milan, Milan, Italy

**Keywords:** bone regeneration 3, CGF 6, graft 2, growth factor 5, PRF 4, scaffold 1

## Abstract

**Objective:** Bone regeneration plays a pivotal role in modern oral surgery, particularly in facilitating successful implant-prosthetic rehabilitation. This systematic review explores the regenerative potential of growth factors, such as platelet-rich fibrin (PRF) and concentrated growth factors (CGFs), when combined with various types of scaffolds in bone augmentation procedures, including guided bone regeneration, split crest, sinus lift (SL), and alveolar ridge preservation.

**Method:** A comprehensive search strategy yielded 18 relevant studies, which were analyzed for bone formation and stabilization outcomes.

**Results:** Results indicate that PRF enhances bone regeneration and stabilization in SL and ridge augmentation procedures, while CGFs facilitate surgical techniques and augment bone. However, some studies did not report significant differences. Growth factors also demonstrate benefits in wound healing, reducing bone resorption, and enhancing socket preservation.

**Conclusion:** Despite valuable insights, further research is needed to comprehensively understand the characteristics of growth factors in various surgical interventions, ensuring informed decision-making in bone regeneration surgery.

## 1. Introduction

In contemporary oral surgery, there is an increasing need to provide more bone volume, aimed at correct implant-prosthetic rehabilitation.

A cone beam computerized tomography evaluation of the width and height of bone in patients who require an implant-supported prosthetic rehabilitation can evidence a not enough quality and amount of bone [1–4]. This can be caused by defects of alveolar bone consequent to trauma or surgical interventions or by normal postextractive alveolar resorption [5–8].

**Figure 1:**
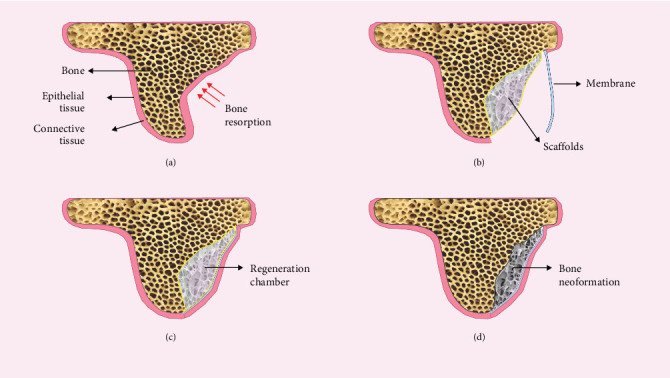
Guided bone regeneration in the alveolar bone defect: (a) alveolar bone defect, (b) guided bone regeneration (GBR), (c) formation of the regeneration chamber after closing the surgical flap, and (d) bone regeneration.

Various techniques of bone regeneration are proposed to enable greater long-term implant-supported rehabilitations. Bone grafting, including autogenous, xenogenous, allogenous, and alloplastic material transplants, can give a sufficient volume of bone to guarantee correct positioning of implant, a predictive osteointegration of these, and successful prosthetic rehabilitation of patients (Figure 1) [9–11]. The relationship between bone formation and resorption is challenging for bone augmentation for implant purposes, considering that healing time for bone stabilization requires 3–4 months [12–14].

One kind of glass called alloplastic graft is derived from a naturally existing mineral named hydroxyapatite. The xenogenous bone transplant is a graft taken from a donor of another species, unlike the allogenous bone graft that is realized using human bone but not collected from the patient that receives the graft [15]. Autogenous bone transplants have been studied mostly for osteoinductive and osteoconductive power and the great biocompatibility of these grafts. Autografts can be derived from intraoral sites, such as the ramus and the symphysis of the mandible and the maxillary tuberosity, or from extraoral donor sites such as the skull, the iliac crest, and the tibia of the human that receives the graft. However, the limits related to donor site bone and the healing process have given way to new bone augmentation techniques, such as the use of growth factors [16].

**Figure 2:**
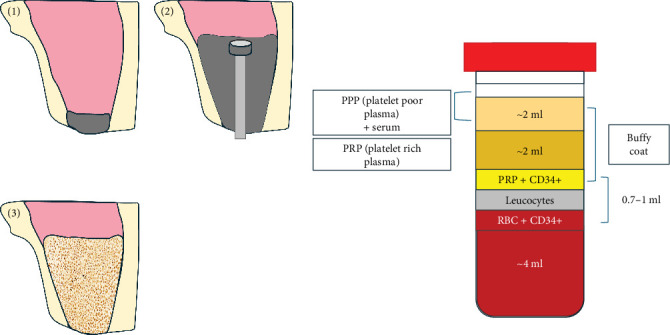
Summer's technique uses a CGF graft for sinus lift. CGF, concentrated growth factor.

In modern tissue engineering, stem cells, growth factors, and scaffolds are three fundamental elements. In oral mouth tissue, regeneration is conditioned by different cells, signal pathways, and matrix interplays [17, 18]. Stem cells are equipped with the so-called “plasticity,” namely these cells replicate continually and can give rise to specialized cell lines [19, 20]. The second element, scaffolds are a sort of microenvironment necessary to achieve cell growth and differentiation, act as a matrix that stimulates cell attachment and migration, and thereby promote the development of organs and tissues [11, 21–23]. Finally, growth factors are proteins that chain receptors inducing the proliferation and differentiation of cells, promoting regeneration of injured tissues [24]. In today's dentistry, the utilization of patients' cells such as platelet concentrates (PCs) derived from patients' blood together with biomaterials as biocompatible scaffolds has shown broad potential in bone regeneration [25]. Dental scaffolds create a framework that must fit into the three-dimensional anatomical defect and temporarily relieve pressure until the expected new bone formation occurs. Blood-derived platelet-rich factors comprise platelet-rich plasma (PRP), platelet-rich fibrin (PRF), plasma-rich growth factors (PRGFs), and concentrated growth factors (CGFs) have acquired significant importance as potential regenerative materials (Figure 2) [20, 26–29].

The aim of this study is to evaluate the regenerative potential of growth factors, such as PRP, PRF, PRGF, and CGF, when used in combination with various types of scaffolds in bone augmentation procedures performed in oral surgery (guided bone regeneration [GBR], split crest, and sinus lift [SL]).

## 2. Materials and Methods

### 2.1. Protocol and Registration

This systematic review was conducted by the standards of the preferred reporting items for systematic reviews and meta-analysis (PRISMA) 2020 statement [30]. The protocol of the review was registered at PROSPERO under the unique number 526206.

### 2.2. Search Processing

The search period started at January 13, 2024, and the last search was carried out at February 26, 2024.

“Bone Regeneration,” “Scaffold,” “Graft,” “Growth Factor,” “CGF,” and “PRF” were the search terms utilized on the databases (PubMed, Web of Science, and Scopus) to select the papers under evaluation, with the Boolean operator “AND” and “OR.” Only content published in English over the previous 5 years (February 2019 to February 2024) was included in the search (Table 1).

### 2.3. Eligibility Criteria

Working in pairs, the reviewers selected pieces that met the following requirements to be included: (1) research involving just human beings; (2) clinical studies.

One of the exclusion criteria was (1) in vitro research. (2) Research on animals (3) case reports; (4) narrative reviews, meta-analyses, and systematic reviews.

Duplicate studies were removed manually.

The review was conducted using the PICO criteria:

• Population: adults, both male and female who needed bone regeneration or preservation.
• Intervention: growth factors and scaffolds used in various oral surgery technique.
• Comparison: procedures of preservation or bone regeneration technique with scaffolds and growth factors.
• Outcome: effectiveness of scaffolds combined with growth factors in bone regeneration or preservation.

### 2.4. Data Processing

Working independently, two reviewers (G.L. and A.D.) used the predetermined inclusion and exclusion criteria to filter the data extracted from each database. Individual choices were hidden from the researchers. Both reviewers' results were converged upon in the final meeting. The complete text was obtained and examined when a reviewer thought an article might be accepted. Both independently and twice, this occurred.

Each qualifying main study's authors and publication date, study type, purpose, materials, and methods, and findings are among the data that were taken from it.

Disagreements between reviewers regarding article selection were resolved through discussion.

### 2.5. Quality Assessment

Two reviewers, G.L. and M.C., evaluated the included papers' quality using the reliable Cochrane risk-of-bias assessment for randomized trials (RoB 2). This test assesses six potential areas of bias: inadequate outcome data, selective reporting, blinding of participants and staff, random sequence generation, allocation concealment, and outcome assessment blinding. A third reviewer (F.I.) was consulted if there was a disagreement and continued until a consensus was reached.

## 3. Results

**Figure 3:**
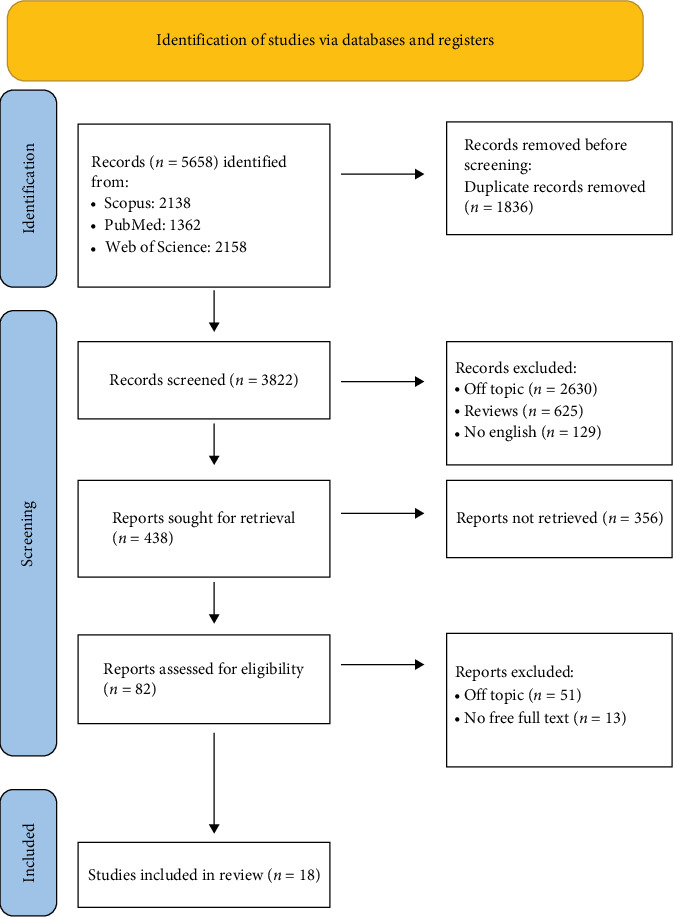
PRISMA flowchart used in this review paper. PRISMA, preferred reporting items for systematic reviews and meta-analysis.

Keyword searches of the Web of Science (2158), Scopus (2138), and PubMed (1362). A total of 5658 articles were found in the databases. After the duplicates were eliminated (1836), 3822 articles were included. Out of these 3822, 2804 studies were disqualified for violating the inclusion criteria that had been previously established. After screening, 18 papers were chosen for this work (Figure 3). Each study's findings are listed in Table 2.

## 4. Discussion

### 4.1. Scaffolds on Bone Augmentation

Scaffolds play a crucial role in bone augmentation and regenerative guidance by providing structural support and mimicking the extracellular matrix, which facilitates cell attachment, proliferation, and differentiation [46–52]. These scaffolds can be made from various materials such as ceramics, polymers, or composites, each with unique properties influencing tissue regeneration. In bone augmentation, scaffolds act as temporary frameworks that support the formation of new bone [53–57]. They provide mechanical stability, prevent soft tissue invasion, and guide the formation of new bone in the desired shape and location [58–64]. The choice of scaffold material depends on factors like biocompatibility, biodegradability, mechanical properties, and ability to promote osteogenic activity [65–70]. Moreover, scaffolds can serve as regenerative guides by directing the behavior of cells and signaling molecules. They can be engineered to release growth factors or bioactive molecules in a controlled manner, promoting tissue regeneration and modulating the healing process. Additionally, scaffold architecture and surface characteristics can influence cell adhesion, migration, and differentiation, further enhancing tissue regeneration. Overall, scaffolds are crucial in bone regeneration by providing a supportive environment for new tissue growth, guiding tissue formation, and facilitating the integration of regenerated tissue with the surrounding native tissue. Continued advancements in scaffold design and fabrication techniques hold promise for improving the efficacy and clinical outcomes of bone augmentation and regenerative medicine approaches [47, 48, 71, 72].

### 4.2. Growth Factors in Oral Surgery and Their Working Mechanisms

Growth factors, including PRP, PRF, platelet-rich growth factor (PRGF), and CGFs, have gained significant attention in oral surgery for their potential to enhance tissue regeneration and wound healing processes [22, 73]. These growth factors are derived from the patient's blood and contain a concentrated mixture of bioactive molecules, primarily platelet-derived growth factors (PDGFs) [74–80]. PRP, PRF, PRGF, and CGF function through similar mechanisms, yet they differ in their preparation methods and composition. Upon activation, platelets release various growth factors such as PDGF, transforming growth factor-beta (TGF-*β*), vascular endothelial growth factor (VEGF), insulin-like growth factor (IGF), and fibroblast growth factor (FGF) [76, 81–89].

The working mechanisms of these growth factors involve:

1. Stimulation of cell proliferation: Growth factors promote the proliferation of various cell types involved in tissue regeneration, including fibroblasts, osteoblasts, and endothelial cells. This leads to accelerated tissue regeneration [90–92].
2. Enhancement of angiogenesis: Growth factors such as VEGF stimulate the creation of new blood vessels (angiogenesis), improving blood supply to the injured area and facilitating the delivery of oxygen and nutrients necessary for tissue healing [93–95].
3. Induction of extracellular matrix production: Growth factors stimulate the synthesis of extracellular matrix components such as collagen, elastin, and glycosaminoglycans, essential for tissue structure and function.
4. Modulation of inflammatory response: Growth factors regulate the inflammatory response by promoting the resolution of inflammation and inhibiting excessive inflammation, thereby creating a favorable environment for tissue healing.

PRP, PRF, PRGF, and CGF differ in their preparation methods and the concentration of growth factors. PRP is prepared by centrifuging whole blood to separate the PRP, while PRF is obtained through a simpler centrifugation process without anticoagulants [96–101]. PRGF involves the use of specific protocols for platelet activation, and CGF is prepared using a more advanced centrifugation technique to obtain a higher concentration of growth factors. In oral surgery, these growth factors are commonly used in procedures such as bone augmentation, socket preservation, sinus elevation, and periodontal therapy to enhance tissue regeneration, promote wound healing, and improve treatment outcomes. Their ability to accelerate healing and reduce complications has made them valuable adjuncts in oral and maxillofacial treatments [41, 50, 102, 103].

### 4.3. PRF and SL

Arumugam et al. [31] evaluated the regenerative outcomes of maxillary sinus augmentation (MAS) using autogenous and alloplastic graft materials, with or without PRF. Patients with edentulism in the posterior maxilla were split in three groups: PRF alone, autogenous bone graft + PRF, and alloplastic bone graft + PRF. Autogenous grafts were picked from mandibular symphysis, ramus, and iliac crest, while alloplastic grafts used *β*-tricalcium phosphate putty. PRF was prepared using Choukroun's Protocol, 9 ml of the patient's blood was drawn a few minutes before placement and collected in vacutainer tubes without anticoagulant. The samples were processed in the centrifuge (REMI Laboratories) at 2700–3000 rotations per minute for 12 min. Both graft types with PRF demonstrated promising bone regeneration for up to 6 months, showing stability and radiodensity maintenance [31, 104, 105].

Another study aimed to evaluate histomorphometric outcomes in employing PRF-free or PRF-containing allograft bone fragments. Individuals having a remnant alveolar bone height of little more than 3 mm and an edentulous maxilla were allocated to groups receiving PRF combined with allografts (intervention) or only allograft particles (control). Bone biopsies were collected 6 months postoperatively to evaluate histologic parameters. The outcomes included newly formed bone area, new bone marrow, residual graft particles (mm^2^), and examined and the width and thickness of the bone at the graft site radiographically. The bone marrow that has just grown was noticeably more in the study group than the control one [32, 106, 107].

de Almeida Malzoni et al. [12] sought to evaluate the effects of mixing deproteinized bovine bone mineral (DBBM) with leukocyte and platelet-rich fibrin (L-PRF) in MSA. Thirty-six maxillary sinuses were grafted with either L-PRF and DBBM or DBBM alone. Cone beam computed tomography (CBCT) and histomorphometric analysis revealed increased new bone in the L-PRF + DBBM group in comparison to the control. Immunohistochemical analysis showed elevated expression of key proteins related to bone creation in the L-PRF + DBBM group. The combined use of L-PRF and DBBM demonstrated improved and accelerated bone regeneration, suggesting it is a promising approach for MSA procedures with early implant placement [12, 108–110].

### 4.4. GF and SL

The purpose of De Almeida Barros Mourão et al.'s [33, 111–113] study was to assess how blood-derived growth factors (BDGFs) affected the performance of carbonated hydroxyapatite (cHA) nanostructured microspheres MSA. In a study involving 10 participants, cHA microspheres were implanted alone or with BDGF in maxillary sinuses. Despite the successful releasing of growth factors from BDGF, no significant improvement in bone repair was observed when cHA was combined with BDGF compared to cHA alone after 6 months. However, BDGF did enhance technical aspects, optimizing surgical procedures and improving biomaterial adaptation in MAS [33, 114, 115].

Chen et al. [34] compared clinical results in MAS with CGF during simultaneous implant placement, either with or without bone grafting, in the maxillary posterior area with a residual bone height (RBH) of 4–6 mm. Findings revealed no significant differences in SL height or bone resorption between groups. Postoperative pain at 14 days was higher in the bone grafting group. Both groups exhibited a total success, confirming the safety and reliability of MAS with CGF, irrespective of bone grafting, for patients with RBH 4–6 mm. The study suggests that CGF, with or without bone graft, yields successful outcomes for MAS in severely atrophic maxillae. It recommends MAS without bone grafting when feasible due to reduced postoperative pain [34, 88, 116–118].

### 4.5. PRF and Ridge Augmentation

The results of dental implants implanted in autogenous bone grafts covered by either PRF membrane or a conventional technique comprising DBBM and a collagen membrane were compared by Hartlev et al. [35, 118–121]. There were 27 patients in the study who were partially edentulous; 14 were in the PRF group and 13 were in the control group. Two implants were lost in the control group (85% survival rate) after a 2-year follow-up, whereas none were lost in the PRF group (100% survival rate). In the control group, implant crown survival was 92%, while in the PRF group, it was 100%. Both groups had healthy soft tissue values; however, the PRF group had a statistically substantially greater peri-implant marginal bone level [36, 122–124].

CBCT scans were done before grafting, as well as at 2 weeks and 6 months postgrafting to evaluate changes in volume. Results showed a mean bone volume loss of 14.7% in the PRF group and 17.8% in the control group, indicating no significant overall difference. However, notable variations were found between regions, with increased resorption in the incisor and canine area in the no-test group. The study concludes that both techniques produce similar results after 6 months, underscoring the importance of considering regional disparities in bone resorption rates [37, 25, 125, 126].

After lateral alveolar ridge augmentation using an autogenous bone graft covered by either PRF membrane or a standard procedure involving a DBBM and a resorbable collagen membrane another study assessed the histologic and histomorphometric bone characteristics, with an emphasis on vitality. The biopsy analysis, performed 6 months postgrafting, revealed comparable low bone vitality in both groups, with no significant differences. No significant differences were observed between the groups [37, 38, 127].

The postoperative pain after lateral ridge augmentation, comparing two groups: one with autogenous bone block grafts covered with PRF membranes, and another with DBBM and a resorbable collagen membrane. Both groups received postoperative medication, and pain was recorded using a visual analog scale (VAS). The results showed generally low postoperative pain scores for all patients, with the PRF group experiencing slightly lower pain perception. However, the difference was only statistically significant on day 1 postoperative. The study highlights the importance of managing pain with effective analgesics and discusses factors such as anesthesia, medications, and patient expectations influencing postoperative pain perception [35, 128, 129].

Işık et al. [39] assessed the success of GBR during implant insertation using bovine-derived xenograft alone and fluid PRF. The primary outcome, augmentation thickness, was measured using CBCT at 6 months postsurgery. The test group showed slightly higher thickness and the bone loss was less than 1 mm over 2 years, and implant survival rates were 100% for both groups. Fluid PRF, in combination with bovine-derived xenograft, is profitable in bone augmentation during implant placement [39, 130, 131].

Desai et al. [40] investigated the effectiveness of PRP in improving bone graft outcomes in 50 patients with alveolar cleft and surgical defects, who were randomly assigned to a study group receiving autologous PRP and the other group without PRP. Hounsfield units measured bone density pre-and post-operatively. Both groups exhibited a significant increase in Hounsfield units at 6 and 12 months, indicating substantial bone regeneration. PRP, a biotechnological application, was shown to enhance bone graft healing in the maxillofacial area. PRP mechanisms emphasize growth factors like PDGF and TGF-*β*, which accelerate bone graft healing through three phases: capillary growth, consolidation, and resorption–remodeling. Utilizing Hounsfield units and 3D CT scans, the research identified statistically significant increases in bone density for both groups, particularly in the study group [40, 132, 133].

Zahedi et al. [41] compared freeze-dried bone allograft (FDBA) alone to FDBA combined with injectable-platelet-rich fibrin (i-PRF) for horizontal ridge augmentation in 41 patients with alveolar ridge defects. Radiographic measurements were taken before and after augmentation, showing no significant width difference but more new bone and fewer residues in the FDBA + i-PRF group. This group exhibited superior bone formation quality, emphasizing i-PRF's benefits like enhanced stability and increased growth factors. In vitro, PRF positively impacted osteogenic markers. The FDBA group had more remaining graft material, potentially due to accelerated regeneration in the i-PRF group. Soft tissue rates were higher in the FDBA group, suggesting reduced stability [41, 134–136].

Abdelfadil and Aboelmaaty [42] evaluated the use of mineralized plasmatic matrix (MPM) for horizontal ridge augmentation, with and without a covering collagen membrane, in 16 edentulous spaces. CBCT images were evaluated for alveolar ridge changes and graft material resorption. Results showed no difference in gained bone width between the groups, with slightly more but not statistically significant resorption in the group without a membrane. The study concluded that MPM can be used for horizontal ridge augmentation without the need for a covering barrier membrane [42, 137–139].

### 4.6. CGF and Ridge Augmentation

Aboelela et al. [9] investigated two techniques for GBR in treating maxillary alveolar ridge resorption for implant rehabilitation. Twenty-eight patients were divided into two groups: one using a mixture of autogenous and DBBM covered by a native collagen membrane, and the other using the same mixture with autologous fibrin glue (AFG) and CGF membrane [112, 140]. There were no significant differences in bone gain between the two techniques, but the CGF membrane was deemed less predictable for GBR. The study highlights the importance of barriers in shielding the augmented site from soft tissue proliferation initially [9, 141–143].

### 4.7. PRF and Socket Preservation

Chary et al. [43, 144, 145] discussed a prospective randomized clinical trial comparing early implant placement with socket preservation using advanced-platelet-rich fibrin (A-PRF) at 6 weeks and 8 weeks postextraction. Two groups of 10 participants each were subjected to atraumatic extraction and preservation of the alveolus. Implants were inserted at 6 and 8 weeks postextraction in a group and in other groups, respectively [76, 146–149]. Results showed significantly higher torque values and bone formation at 8 weeks compared to 6 weeks, indicating the efficacy of A-PRF in accelerating bone regeneration. The study highlights the advantages of A-PRF in promoting soft tissue regeneration and bone formation, thus supporting early implant placement protocols at 8 weeks when compared to 6 weeks [43, 150–152].

Azangookhiavi et al. [44] aimed to compare the effectiveness of FDBAs and PRF for socket preservation after tooth extraction. Thirty-two patients underwent extraction of nonmolar teeth and were split into two groups: FDBA and PRF. After 12 weeks, changes in bone dimensions were assessed. Both groups showed a significant reduction in ridge width, with PRF yielding similar results to FDBA. The bone height changes were not significant. Although the FDBA group had less bone resorption, PRF proved effective in reducing ridge resorption compared to baseline. PRF application without graft materials provided optimal ridge preservation, making it a cost-effective alternative to FDBA [44, 153, 154].

Abaza et al. [45] aimed to compare i-PRF with hyaluronic acid (HA) combined with xenografts for alveolar ridge preservation (ARP) after tooth extraction. In total, 36 patients had ARP and received one of three treatments: i-PRF with xenografts, HA with xenografts, or xenografts alone. CBCT scans and clinical assessments were conducted at 4 months and 1 year postsurgery, and histological evaluation was performed at 4 months. Results showed that the HA group had the highest radiographic bone gain and superior histological outcomes compared to the i-PRF and other groups. Additionally, i-PRF improved soft tissue thickness. HA combined with xenografts demonstrated superior bone preservation outcomes compared to i-PRF and control groups, both clinically and radiographically. Overall, HA showed beneficial effects on ARP compared to i-PRF for bone preservation and maturation. However, i-PRF enhanced soft tissue thickness [45, 155, 156].

### 4.8. CGF and Socket Preservation

Ma et al. [8] assessed the effects of CGF on ARP after posterior tooth extraction. Fifty patients were assigned to CGF treatment or no treatment. Healing scores, CBCT scans, and histological analyses were conducted. Results showed improved healing scores at 10 days postextraction and reduced vertical and horizontal bone resorption with CGF treatment. Micro-CT analyses indicated better bone mineral density and microarchitecture in the CGF group. One year after implant restoration, both groups showed 100% success rates. CGF application may effectively reduce bone resorption and promote new bone regeneration postextraction [8, 157, 158].

Overall, the study suggests PRP's promise in tissue engineering, enhancing autologous bone graft osteogenic potential and reducing postoperative bone resorption, making it a valuable tool in oral and maxillofacial surgery. The study group demonstrated greater regeneration, supported by volume ratio and statistical significance.

## 5. Conclusions

In conclusion, the studies reviewed demonstrate how scaffolds act as a framework in regeneration bone procedures and highlight the growing importance of growth factors in bone augmentation and wound healing. PRF appears to enhance bone regeneration and stabilization both in SL and ridge augmentation, while CGF can facilitate surgical techniques and enhance bone augmentation. However, some studies do not show significant differences. For what concerns wound healing, the use of growth factors guarantees less bone resorption and better socket preservation. Despite the important insights offered by this review, anyway, several limitations must be considered. For example, this systematic review does not comprehend studies conducted on the use of growth factors combined with scaffolds in regeneration procedures in the field of periodontal surgery and endodontic surgery. Moreover, larger scale epidemiological investigations are important to validate the findings cited. Future research should analyze other fields of application of growth factors to provide a more comprehensive understanding and facilitate better comparisons of characteristics of the factors studied. By filling these knowledge gaps, we can choose with greater awareness the optimal surgical technique for bone regeneration.

## Figures and Tables

**Table 1:** Database search indicators.

Article screening strategy	Database: Scopus, Web of Science and PubMed
	Keywords: (A) “Bone Regeneration”; (B) “Scaffold”; (C) “Graft”; (D) “Growth Factor”; (E) “CGF”; (F) “PRF”
	Boolean variable: “AND” and “OR”
	Timespan: 2019–2024
	Language: English

**Table 2:** Examined articles.

Authors	Type of the study	Age and number of participants	Aim of the study	Materials and clinical data	Results and percentages
Arumugam et al. [31]	RCT (randomized controlled trial)	14 patients between 20 and 60 years	Evaluate the regeneration of bone and compare the efficacy of PRF in bone regeneration for sinus augmentation surgery	Group I: PRF as a stand-alone agent; Group II: Autogenous bone graft + PRF; Group III: Alloplastic bone graft (Tricalcium phosphate putty) + PRF	PRF used with alloplastic or autogenous graft augmented bone regeneration by providing growth factors for bone regeneration

Shiezadeh et al. [32]	RCT	20 patients with study group (42.7 ± 5.79 years) and with control B (40.3 ± 4.83 years)	Evaluate and compare the histomorphometric outcomes in MAS using allograft bone particles with or without PRF	In the study group MAS was performed using PRF combined with bone allografts, while in the other groups, only allograft particles were used	In the study group, bone marrow was more and residual graft particles were less than in the other group

de Almeida Malzoni et al. [12]	RCT	24 patients between 26 and 69 years	Evaluate the formation of bone and compare the efficacy of L-PRF in bone regeneration for sinus augmentation surgery	Group I: With a blend of leukocyte and platelet-rich fibrin (L-PRF) along with DBBM. Group II: Exclusively with DBBM. Dental implants were put in the experimental group following two assessment periods at 4 and 8 months. Cone beam computed tomography (CBCT) scans were conducted 1 week postsurgery (T1) and before implant placement (T2)	L-PRF can be useful in combination with DBBM for MAS allowing the installation of appropriate-length implants in a shorter time

de Almeida Barros Mourão et al. [33]	RCT	10 patients between 46 and 67 years	Evaluate if BDGF improved the efficacy of a modified carbonated calcium phosphate biomaterial in MAS	20 MAS using nanostructured carbonated hydroxyapatite (cHA) or with BDGF were exanimated after 180 days with CBCT	Blood-derived growth factors did not improve bone repair when associated with cHA in MAS

Chen et al. [34]	Retrospective study	44 patients >18 years	Evaluate MAS with CGF and simultaneous implant inserted with or without bone graft in the maxillary posterior region	Group A, with bone grafting; Group B, without bone grafting had MAS combined with CGF and the simultaneous placement implants	There were no differences between the two groups, the implant success was 100% with or without bone grafting

Hartlev et al. [35, 36]	RCT	27 patients between 23 and 72 years	Evaluate the clinical performance of implants placed in sites previously augmented with autogenous bone grafts covered by either PRF and membrane (PRF group) or with autogenous bone graft and DBBM with a collagen membrane	Patients for dental implant placement was embedded. They had final clinical and radiographic follow-up to control outcomes	Either group showed similar outcomes

Hartlev et al. [37]	RCT	27 patients between 23 and 69 years	Evaluate the changes using autogenous bone graft covered by PRF membrane or a DBBM and a collagen membrane	The edentulous patients were examination with CBCT before treatment and at 2 weeks and 6 months after treatment	Either group showed similar outcomes at 6 months

Hartlev et al. [38]	RCT	27 patients > 20 years	Evaluate histologic and histomorphometric bone characteristics with a focus on vitality after lateral alveolar ridge augmentation using a block covered by either a PRF membrane (test group) or a standard procedure involving coverage of the bone block with a DBBM and a resorbable collagen membrane	In patients with an indication for bone block augmentation before implant insertion was performed a biopsy of augmented bone 6 months after	A comparable augmented bone was noted in both groups

Hartlev et al. [35, 36]	RCT	27 patients >20 years	Evaluate pain after bone augmentation. Autogenous bone was covered with PRF membranes or DBBM and collagen membrane	The pain was evaluated by the patient on the visual analog scale (VAS), 1 h on the day of treatment and after 7 days	Adding PRF membranes resulted in slightly lower pain perception, although a statistically significant difference between the 2 groups was identified on day 1 postoperative

Işık et al. [39]	RCT	40 patients > 18 years of age	Evaluate the augmentation success after guided bone regeneration (GBR) carried out simultaneously with implant placement using bovine-derived xenograft alone and in combination with fluid PRF	After implant placement, GBR procedures were performed using fluid PRF-enriched bovine-derived xenograft (for the test group) and with bovine-derived xenograft alone (for the control group)	The bone loss was less than 1 mm for both groups during the 2 years of follow-up

Desai et al. [40]	Prospective study	50 patients between 8 and 50 years	Evaluate the changes with bone grafts for defects after the removal of osteolytic jaw lesions	The patients were split in a PRP group and a group without PRP. Bone density was calculated for both the groups	There was greater bone formation in the PRP group

Zahedi et al. [41]	RCT	41 patients between 24 and 71 years	Evaluate the effectiveness of bone augmentation using FDBA in combination with injectable-platelet-rich fibrin (i-PRF) versus FDBA alone	After the treatment, biopsies were performed on the augmented bone and these were examined histologically and histomorphometrically	In the FDBA + i-PRF group there was new quality bone, less connective tissue, and less particles

Abdelfadil and Aboelmaaty [42]	Retrospective study	15 patients, >18 years of age	Evaluate horizontal ridge augmentation using mineralized plasmatic matrix (MPM) with and without a coverage membrane	MPM was used for horizontal ridge augmentation with and without a covering collagen membrane (groups 1 and 2)	There was no difference between both groups in the gained bone width

Aboelela et al. [9]	Single-centered, prospective, controlled, parallel armed randomized clinical trial	28 patients, >18 years of age	Evaluated the bone regeneration using a 1:1 mixture of autogenous particulate and an organic bovine bone mineral (ABBM)	In the control group, was used native collagen membrane. In the study group, it was mixed with autologous fibrin glue (AFG) and it was covered by CGF membrane	Between the two groups, there was no statistically significant difference regarding bone gain

Chary et al. [43]	RCT	10 patients, between 20 and 50 years	Evaluate the use of Advanced platelet-rich fibrin (A-PRF), in accelerating bone regeneration potential. Early implant placement with a limited healing period, along with A-PRF, can be beneficial	Early implant placement in sockets preserved using A-PRF at 6 weeks and 8 weeks of postextraction, in group A and group B, respectively. The insertion torque values were recorded during implant placement	*T*-test for torque values indicated a significantly higher torque value at 8 weeks

Azangookhiavi et al. [44]	RCT	32 patients, >18 years of age	Evaluate the clinical application of freeze-dried bone allografts (FDBA) and PRF for ARP after tooth extraction	Tooth sockets were replenished with either FDBA or PRF. Bone regeneration was evaluated for changes in horizontal and vertical bone dimensions after 12 weeks of implant insertion	The use of PRF in extraction sockets yielded similar results to FDBA

Abaza et al. [45]	RCT	36 patients, between 20 and 50 years	Evaluate the effectiveness of injectable-platelet-rich fibrin (i-PRF) versus hyaluronic acid (HA) in combination with xenografts for ARP after tooth extraction	i-PRF with xenografts HA with xenografts, or xenografts alone. ARP was performed after extraction and implants were inserted. CBCT scans were done before and 4 months postsurgery	HA with xenografts showed new bone formation than the other groups

Ma et al. [8]	RCT	50 patients, >20 years	Evaluate the impact of autologous concentrated growth factors (CGFs) on alveolar ridge absorption and osteogenesis following posterior tooth extraction	The extraction sockets were treated with CGF or no treatment. CBCT scans were used to assess bone changes	The use of CGF after extraction may reduce vertical and horizontal bone resorption and promote new bone formation

## Data Availability

Data sharing is not applicable to this article as no new data were created or analyzed in this study.

## Nomenclature

ACS: Absorbable collagen sponge
A-PRF: Advanced-platelet-rich fibrin
ARP: Alveolar ridge preservation
BDGFs: Blood-derived growth factors
CBCT: Cone beam computed tomography
CGFs: Concentrated growth factors
cHA: Carbonated hydroxyapatite
DBBM: Deproteinized bovine bone mineral
FDBAs: Freeze-dried bone allografts
FGF: Fibroblast growth factor
GBR: Guided bone regeneration
HA: Hyaluronic acid
IGF: Insulin-like growth factor
i-PRF: Injectable-platelet-rich fibrin
L-PRF: Leukocyte and platelet-rich fibrin
MAS: Maxillary sinus augmentation
PDGF: Platelet-derived growth factor
PRF: Platelet-rich fibrin
PRGF: Platelet-rich growth factor
PRP: Platelet-rich plasma
RBH: Residual bone height
RCT: Randomized controlled trial
rhBMP-2: Recombinant human bone morphogenetic protein-2
SL: Sinus lift
TGF-*β:*: Transforming growth factor-beta
VAS: Visual analog scale
VEGF: Vascular endothelial growth factor.
